# Combining art and science in healthcare

**DOI:** 10.1080/17571472.2016.1169746

**Published:** 2016-04-04

**Authors:** Paul Thomas

**Affiliations:** ^a^ LJPC

Science can only ascertain what is, but not what should be. Albert Einstein

This Issue of LJPC contains two papers that provide new insight into the old puzzle of how to combine Art and Science in health and health care.

Through a discussion of music and metaphor, Hallam and Whitehouse show that Art and Science provide complementary insights into how to live a healthy life. Art is good at forward-looking co-creative activities. Science is good at the more retrospective task of working out what actually worked – for example whether an antibiotic defeated a bacterium in an experiment. Both are needed.

A healthy person moves their life story forwards in a healthy way by creatively interacting within a complex world. Toon explains this well – health is an *aliveness of spirit* that enables someone to develop a *flourishing life story*.[1] In a similar vein, Antonovski has argued that being healthy means *rising above adversity*.[2] Health is an *active* thing that helps someone to live in the moment and turn bad things into good. Of course disease can get in the way of health, but the absence of disease does NOT make someone healthy. Indeed, the onset of disease often has the effect of waking someone up to the need to start living their life in a healthier way. Health care needs to help people to be healthy as well as cure their illnesses.

Hallam’s paper has already caught readers’ attention, achieving 240 downloads merely in the time it has been sitting on the LJPC website waiting for publication. It shows a range of health benefits to people aged over 50 when they take part in musical activities. But look at what those benefits are:

• Social benefits – a sense of belonging.
• Cognitive benefits – improved concentration and memory.
• Health benefits – improved mobility, vitality and feelings of rejuvenation.
• Emotional benefits – protection against stress and depression and the development of a sense of purpose.

These are the same things that we need to feel healthy at any stage of life; the same things that health care workers need to do their everyday work well; the same things that build the trusted relationships and teams, needed for integrated care.

Hallam has previously published evidence that taking part in music improves the academic achievement of children.[3] This work is now being used in the UK to argue that music should be a part of every child’s school experience. In this LJPC paper, she now presents evidence that music is also good for your health. Messages for LJPC readers are that General Practice waiting rooms should advertise ways for patients to take part in musical activities; primary care workers should advise those who are depressed or anxious to take up music. Health care professionals should heed their own advise, and get musical.

One obstacle to exploring how music improves health is that the approach to science preferred by medicine is poor at seeing co-creative activities. Take a jazz band for example; quality music is not simply a set of individual notes or chords but a sequence that speaks to a particular listener. Quality improvisation comes from creative interaction between participants from which emerges such a new sequence. Similarly, a flourishing narrative comes when someone creatively adapts to life’s opportunities.

Co-creative activity is difficult to see through the lens of a medical science which relies heavily on an approach to knowledge generation, termed *positivism*. ‘Naïve positivism’ examines factors one at a time as isolated particles that interact in direct, ‘linear’ ways with other particles. It uses a mechanistic metaphor of the *world as a laboratory, or a machine* within which ‘causes’ have predictable ‘outcomes’. This encourages the idea that the world can be simply controlled. This approach helps to see if a treatment will defeat a disease, but it does not help to understand complex and co-creative activities that affect different people differently. It cannot explain how music can heal.

Whitehouse suggests that ecological metaphors help to understand co-creative phenomena by: *‘blending ideas from often disconnected corners of our minds … and sharing novel ideas throughout communities of thought’ …. Metaphors* [differ from scientific theories by being] *intergenerative* – *meaning innovating through integration or blending and going ‘between’ to go ‘beyond’.* LJPC readers will agree with Whitehouse that this is important because *‘stories and metaphors matter in the health of individuals and communities, and performance art can contribute to opening minds and hearts’.*

Using the metaphor of a tree to expand his idea, he describes how thinking of ‘*the tree of life’* or ‘the *brain as a tree’* leads to expectations that life and brains are dynamic, co-evolving wholes, quite different from the metaphor of life or a brain as a machine. Ecological metaphors like this provide a vision of the whole and ways to engage with it, rather than a simple protocol to follow. Participants need to interpret their actions from these metaphors in ways that make sense to their personal journeys – their tunes.

Whitehouse’s idea that metaphors can enable forward-looking, creative exploration of the future chimes with ideas from organisational behaviour and complexity research about how to overcome the limitations of ‘*naïve positivism’*.

From the organisational behaviour literature, Morgan describes how a *machine metaphor* gives rise to a controlling type of organisation – like a bureaucracy which is good when things are simple and stable; while a *living organism metaphor* gives rise to *open systems* or *learning organisations* – good when things are complex and constantly changing.[4] He then offers six other metaphors – Organisations as Brains, Cultures, Political Systems, Psychic Prisons, Flux & Transformation, and Domination each of which leads to quite different implications for policy and evaluation.

From the research literature, Guba describes three paradigms of inquiry that have emerged out of naïve positivism – post-*positivism, critical theory* and *constructivism*.[5] Post-positivism examines individual facts (or musical notes), but acknowledges that the world (the tunes) is more than those facts. *Critical theory* looks at the hidden connections that exist between different factors – for example networks of relationships across organisational boundaries (or musicians playing in tune). *Constructivism* looks at co-emergence from creative interaction – for example new health care services (new tunes).

The metaphor of *healthcare as music* suggests that Science and Art can be combined by oscillating between what is, and what should be. That jazz band again – positivist science can precisely document what notes have been played so they can be copied by others; it can provide a beat that helps players to stay in time. But art is needed to feel your way into the tune, listen with appreciation to others and to yourself, and to ‘play’ with fellow musicians in ways that co-create something that is more than the sum of the parts.

So-called ‘Soft’, co-creative, relationship-building Art, and ‘Hard’, linear, controlling Science have been unhelpfully separated in health care in recent years. We need new ways to re-entwine them to be mutually enhancing. Researchers need to more often use multiple methods inquiry. Primary care clinicians need to encourage patients to ‘play’ as a way of moving their life stories forwards in healthy ways. Policy-makers need to devise infrastructure that enables practitioners and managers to creatively work across organisational and disciplinary boundaries, building teams and shared vision for integrated care.

Paul Thomas Paul.thomas7@nhs.net
